# Lightning Behaviour during the COVID-19 Pandemic

**DOI:** 10.12688/f1000research.70650.2

**Published:** 2021-10-27

**Authors:** Fazandra Yusfiandika, Siow Chun Lim, Chandima Gomes, Aravind Chockalingam, Lee Cheng Pay

**Affiliations:** 1 Faculty of Engineering, Multimedia University, Cyberjaya, Selangor, 63100, Malaysia; 2 School of Electrical & Information Engineering, University of the Witwatersrand, Johannesburg, South Africa; 3 School of Computer Science & Engineering, Taylor's University, Subang Jaya, Selangor, Malaysia; 4 Electrical Engineering, Duriane Professionals, Puchong, Selangor, Malaysia

**Keywords:** lightning, air temperature, relative humidity, COVID-19, thunderstorm

## Abstract

Background

COVID-19 has drastically dampened human activities since early 2020. Studies have shown that this has resulted in changes in air temperature and humidity. Since lightning activities are dependent on air temperature and humidity, this study is conducted to evaluate the correlation between the intensity of lightning activities with the atmospheric changes, and investigates the changes, in lightning activities due to atmospheric changes during the COVID-19 pandemic.

Methods

The hypothesis was tested through a t-test and Pearson’s correlation study. The variation trend of lightning strikes count (LSC) in Europe and Oceania during the five months COVID-19 lockdown period (March – July) compared to the same period in the previous five years from 2015 to 2019 is investigated.

Results

Statistical analysis shows the LSC in Europe and Oceania during the lockdown period dropped significantly by more than 50% and 44% respectively compared to the same period in previous five years. Furthermore, LSC was found to be positively correlated with air temperature and relative humidity in Europe. However, in Oceania, LSC seems to be only positively correlated with air temperature but negatively correlated with relative humidity.

Conclusions

This study seems to suggest that lightning activities have significantly changed during this pandemic due to reduction in human activities.

## Introduction

Many countries have enforced lockdown since the beginning of the COVID-19 pandemic. ^1- 3^ Energy-intensive human activities such as travelling and the hospitality sector were drastically reduced resulting in reduced emissions of greenhouse gases. ^4^ The global ${\mathrm{CO}}_{2}$ emission is estimated to drop by 8.8% (−1551 Mt) in the first half-year of 2020 compared to the same period in 2019. Moreover, almost 18% of ${\mathrm{CO}}_{2}$ emissions in recent years were produced from ground transportation. ^5^

With the exception of the preliminary findings by Jones et al., ^6^ the general expectation by researchers is that the trend of temperature is expected to be reduced due to the reduction in CO _2_. A significant positive correlation between the atmospheric temperature and ${\mathrm{CO}}_{2}\;$ emission is reported in. ^7^ Furthermore, COVID-19 lockdown has caused micro-climate changes such as localized variations in air temperature and relative humidity. ^8^ The pandemic is also having an effect on ${\mathrm{NO}}_{X}$ , causing a decline that could possibly lead to short-term cooling. ^9^ Air humidity will also be affected as global warming are dependent on both temperature and humidity. ^10^ This reduction in human activities could also result in drop in aerosol level globally. This reduction in human activities could also result in drop in aerosol level globally. The result of lockdown has disrupted human and industrial activities around the world. The lockdown generally leads to a notable change in carbon dioxide (CO _2_), temperature, and humidity. However, the reduction in human activities may reduce greenhouse gases and may result in a drop in global temperature. The study by Singh et al. found that implementation of lockdown reduced the percentage of temperature and may mitigate the pace of climate change in the future. ^8^

Lightning, a natural atmospheric discharge, is affected by various environmental factors. Lightning brings about hazards to human life and appropriate risk assessment has to be conducted for any habitable structure. ^11, 12^ Atmospheric variables such as climate change, humidity, aerosol level, and wind motion can affect the cloud charge distribution, electric field and threshold electromagnetic fields that give rise to air breakdown. It is predicted that lightning may strike more frequently as a result of the ongoing climate change. ^13^ The lightning intensity may also increase due to the high greenhouse gases in the atmosphere. However, study by Finney et al. stated that many previous studies found a positive correlation between lightning and temperature, and one previous study that found lightning decreases with an increase in temperature. ^14^ This may explain that such relationships become highly uncertain on longer timescales. When warm, wet air rises into the cold air, thunderstorms form. As the warm air cools, moisture in the form of water vapour condenses into water droplets, a process known as condensation. Cooled air descends through the atmosphere, warms up, and rises again. A convection cell is a circuit of rising and descending air. A cloud will form if this happens in a small amount. A thunderstorm can arise if this happens with a lot of air and moisture. The presence of high air temperature and high relative humidity can quickly rise and cause powerful updrafts. These updrafts carried water droplets and quickly froze and collided with ice crystals and graupel, causing the charge transfer process.

Lightning could also be triggered by aerosols released by industrial processes and transportation activities. ^15, 16^ Aerosol could affect lightning activity through modification of cloud micro-physics. Aerosol particles serve as cloud condensation nuclei and ice nuclei, and the amount of this particles could affect the formation of cloud droplets and ice particles. More aerosol will suppress the coalescence and making the average size of cloud droplet to be reduced as well as inhibiting precipitation. Therefore, the process enables the water droplets to rise further to upper layers of the clouds and may enhance the lightning processes. During the lockdown period, many industrial sectors stopped operating. Thus, human activities have considerably reduced during the COVID-19 pandemic which may affect the rate of lightning. Lightning ground flash density tends to increase with drier and warmer surface air. ^17^ Furthermore, the frequency of thunderstorms shows a major peak during summer time. ^19^ Previous studies have also found a strong relationship between relative humidity and lightning occurrence. ^19, 20^ Studies from Pinto Neto et al., ^21^ and Perez-Invernon et al., ^22^ found lightning intensity decreased drastically during lockdown period compared to previous year. This is due to the average value of aerosol which played a major role in lightning events have dropped significantly during lockdown period. Last but not least, Chowduri et al. also concurred that the reduction in particulate matter and aerosol concentration bears strong correlation with the reduction in lightning activity in Kolkata during the COVID-19 lockdown. ^23^

Hence, it is of interest to investigate the correlation between the environmental changes that happened during the period of COVID-19 related restriction of human activities and the lightning occurrence density. This study is an attempt to analyse this situation. This study investigates the trend of five months of lightning occurring from March to July in 2020 compared with the same period (March-July) in 2015-2019 in Europe and Oceania. The outcomes of this work could yield interesting insights into the correlation between human activities and lightning frequency.

## Methods

### Overview

Lightning stroke counts (LSC) and two atmospheric factors namely air temperature and relative humidity are considered as the variables in this study. The relationship between LSC with respect to air temperature and relative humidity will be statistically analysed via the dependent t-test and Pearson correlational studies.

### Data description

From March until July in Europe and Oceania, the total LSC from the year 2015 to 2020 were extracted from LightningMaps.org. ^24^ LightningMaps.org provides historical data of LSC and has been widely used in previous studies. ^25, 26^ The distribution of LSC data is presented in Tables 1 and 2.

**Table 1.** LSC in Europe (2015-2020).

Month	Day	Year					
		2015	2016	2017	2018	2019	2020
March	1-10	35941	94976	85347	80813	11992 3	88282
	11-20	92900	98426	17198	127512	112124	69633
	21-31	241800	57226	70447	122112	124324	130382
April	1-10	135720	147551	319021	342496	461290	67008
	11-20	127720	303901	137021	369496	358290	271908
	21-30	388220	112051	42021	1030496	739790	299408
May	1-10	695659	337627	475664	1365371	497596	426035
	11-20	982159	572627	1097164	1248870	1101096	432035
	21-31	827659	1956427	1807664	2232870	949296	481535
June	1-10	1999717	1774966	1753743	3413850	1808843	858421
	11-20	2140519	1423466	1058744	2299751	2531043	1080921
	21-30	636219	2987966	3270243	1907351	1555843	942421
July	1-10	2506871	1308306	2164302	2129821	2278580	1056066
	11-20	1271672	2028806	2014302	2183320	1589581	663066
	21-31	1610671	3076806	3005001	2908220	2051581	1207066

**Table 2.** LSC in Oceania (2015-2020).

Month	Day	Year					
		2015	2016	2017	2018	2019	2020
March	1-10	53851	114529	143439	160457	304771	107194
	11-20	104251	138479	255589	98857	497771	99195
	21-31	119252	78379	166239	177507	393771	183794
April	1-10	175097	49857	95565	162905	204339	156121
	11-20	147197	50706	77865	252705	120840	139171
	21-30	167797	56506	94165	127106	171840	98270
May	1-10	85097	49651	91015	94666	149871	122090
	11-20	35348	66301	92465	125166	159671	88940
	21-31	21098	64000	191704	139816	233271	54040
June	1-10	17875	16396	116735	57739	141588	96783
	11-20	14075	20386	82686	93488	92288	60583
	21-30	12475	52596	45036	114838	72338	73683
July	1-10	17891	32009	59424	104417	60083	134932
	11-20	69291	51699	92524	98017	90032	89333
	21-31	42891	40409	147474	135016	147382	82382

The air temperature and relative humidity data from March until July in Europe and Oceania from year 2020 are extracted from the Physical Sciences Laboratory using Panoply Version 4.12.0. ^27^ Europe is divided into seven sub-regions such as North Europe, West Europe, Central Europe, East Europe, South Europe, Southeast Europe, and the British Isles. After that, eight points (57.5°N, 10.0°E; 42.5°N, 12.5°E; 50.0°N, 25.0°E; 50.0°N, 5.0°E; 50.0°N, 10.0°E; 50.0°N, 20.0°E; 52.5°N, 0.0°; 42.5°N, 22.5°E) of around the sub-regions of Europe were selected in this study. For the Oceania region, five points (−12.5°N, 132.5°E; −37.5°N, 142.5°E; −27.5°N, 152.5°E; −30.0°N, 115.0°E; −27.5°N, 135.0°E) covering the North, South, East and West of Australia; Three points (−37.5°N, 175°E; −45.0°N, 167.5°E; −42.5°N, 170.0°E) covering the North, South and Centre of New Zealand; one point (−10.0°N, 147.5°E) from Papua were considered. Tables 3 and 4 show the average value of air temperature and relative humidity in Europe and Oceania in year 2020.

**Table 3.** Air temperature and relative humidity in 2020 (Europe).

Month	Day	Average air temperature (°C)	Average relative humidity (%)
March	1-10	7.36	77.96
	11-20	8.77	73.63
	21-31	10.20	69.12
April	1-10	11.39	67.81
	11-20	12.22	69.38
	21-30	13.04	71.16
May	1-10	14.28	73.47
	11-20	15.33	76.71
	21-31	17.57	80.11
June	1-10	18.69	80.59
	11-20	18.99	78.23
	21-30	19.56	75.71
July	1-10	20.09	74.82
	11-20	20.66	75.25
	21-31	21.23	75.75

**Table 4.** Air temperature and relative humidity in 2020 (Oceania).

Month	Day	Average air temperature (°C)	Average relative humidity (%)
March	1-10	21.18	72.82
	11-20	20.65	73.04
	21-31	20.13	73.34
April	1-10	19.38	73.81
	11-20	18.37	74.33
	21-30	17.38	74.74
May	1-10	16.67	75.27
	11-20	16.33	75.86
	21-31	16.01	76.46
June	1-10	15.69	76.58
	11-20	15.34	76.17
	21-30	15.01	75.79
July	1-10	14.98	75.22
	11-20	15.32	74.53
	21-31	15.65	73.82

### Statistical approach

A dependent t-test is was conducted using Microsoft Excel 2016 ( Microsoft Excel, RRID:SCR_016137) to determine whether there is a statistically significant difference between the LSC during the lockdown period in the year 2020 and the LSC in the same period (March-July) in year 2015 until 2019. The LSC is measured from a single population (Europe or Oceania) and two different timelines (before and during). Period A represents the lightning activities before lockdown period i.e. March to July in year 2015 to 2019. Period B represents the lightning activities during the lockdown i.e. March to July in the year 2020.

The t-test is conducted by comparing the data from Period B and Period A. The null hypothesis, $H_{0}$ and the alternative hypothesis, $H_{a}$ is defined as below:

H _0_: There is no significant difference in lightning frequency in between Period A and Period B.

H _a_: There is a significant difference in lightning frequency in between Period A and Period B.

The confidence level of 95% at a significant level, α=0.05 is used. This approach tests the hypothesis and calculates the probability of determining whether there is evidence to reject the null hypothesis. When the *P* value < 0.05, the null hypothesis is rejected, and vice versa.

Next, the Pearson correlation coefficient is used to evaluate the correlation between the frequency of lightning activities with the atmospheric changes. The Pearson’s correlation coefficient, r, is computed to measure the strength of the relationship between total lightning strikes, air temperature, and relative humidity in Period B.

Furthermore, the correlation between the variables was analysed using regression and correlation analyses. The significant level, P value can be obtained from the regression data analysis. The null hypothesis, $H_{0}$ and the alternative hypothesis, $H_{a}$ is defined as below:

Null hypothesis, H _0_: P = 0, There is no significant relationship between lightning strikes with air temperature or relative humidity.

Alternative hypothesis, H _A_: P ≠ 0, There is a significant relationship between lightning strikes with air temperature or relative humidity

By using the P-value method ( α=0.05 ), the decision on rejection or acceptance of the null hypothesis can be made. There is sufficient evidence to conclude that there is a significant correlation between lightning strikes and air temperature or relative humidity as the correlation coefficient is significantly different from zero. Exact P values and the mean value of lightning strikes from May to July are provided in Table 5.

**Table 5.** t-test results comparing lightning strikes in 2020 with previous years in Europe.

**Comparison of lightning strikes in 2020 and 2019**			
**Year**	**Mean**	**P-value**	**Decision**
2020	538279	<.001	Reject $H_{0}$
2019	1085280		
**Comparison of lightning strikes in 2020 and 2018**			
**Year**	**Mean**	**P-value**	**Decision**
2020	538279	<.001	Reject $H_{0}$
2018	1450823		
**Comparison of lightning strikes in 2020 and 2017**			
**Year**	**Mean**	**P-value**	**Decision**
2020	538279	.011	Reject $H_{0}$
2017	1154525		
**Comparison of lightning strikes in 2020 and 2016**			
**Year**	**Mean**	**P-value**	**Decision**
2020	538279	.016	Reject $H_{0}$
2016	1085409		
**Comparison of lightning strikes in 2020 and 2015**			
**Year**	**Mean**	**P-value**	**Decision**
2020	538279	.013	Reject $H_{0}$
2015	912896		

## Results and discussion

### Europe

**Figure 1.**
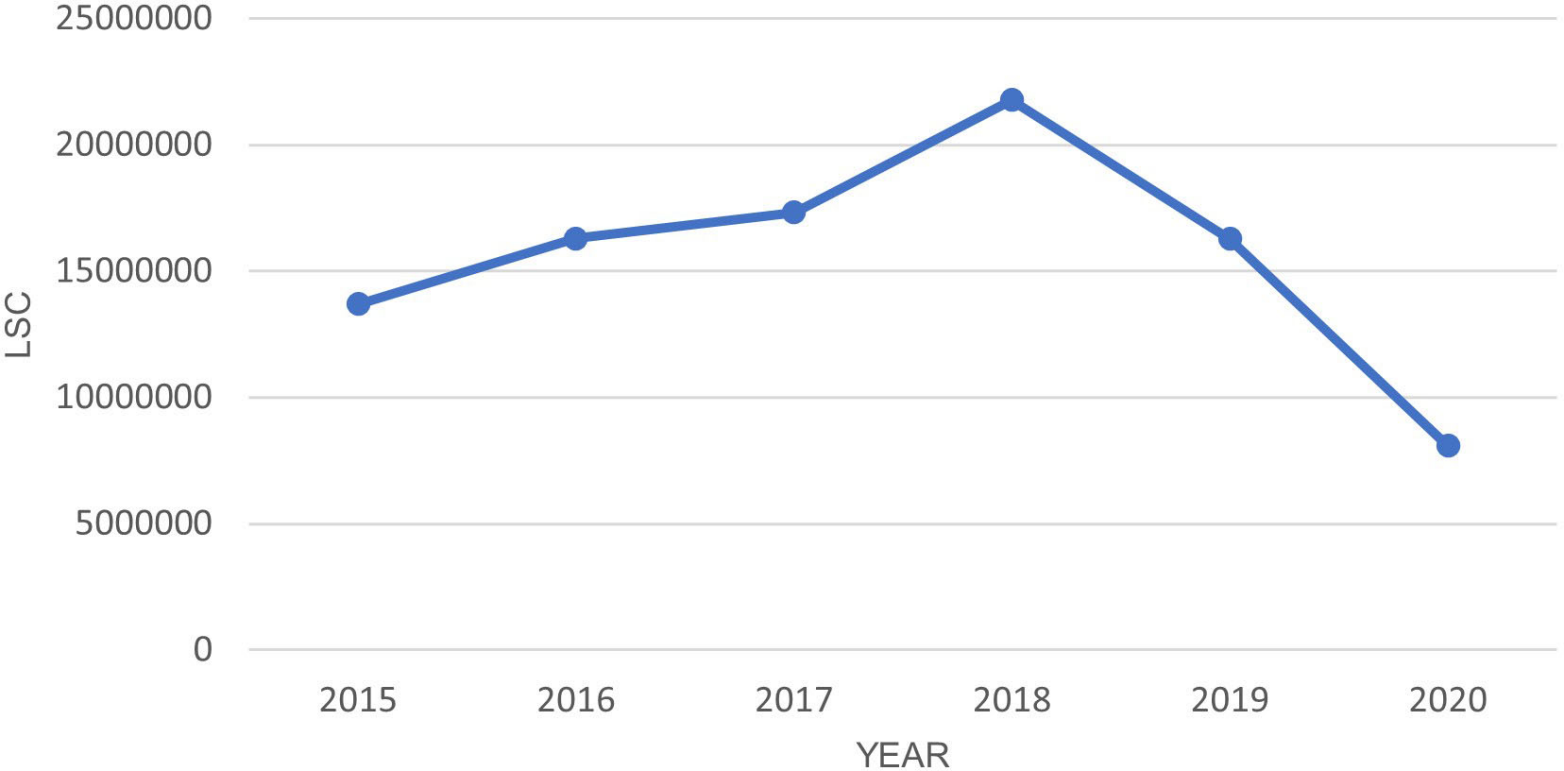
LSC in Europe from March-July in year 2015–2020.

Figure 1 shows the LSC has dropped significantly in the year 2020 when the lockdown started. The dependent t-test shows a statistically significant (P-value <0.05) difference between 2020 and each previous year as shown in Table 5. Notably, LSC in Europe during the five-month lockdown period were reduced by more than 50% compared to the same period in the year 2019, 2018, and 2017.

**Figure 2.**
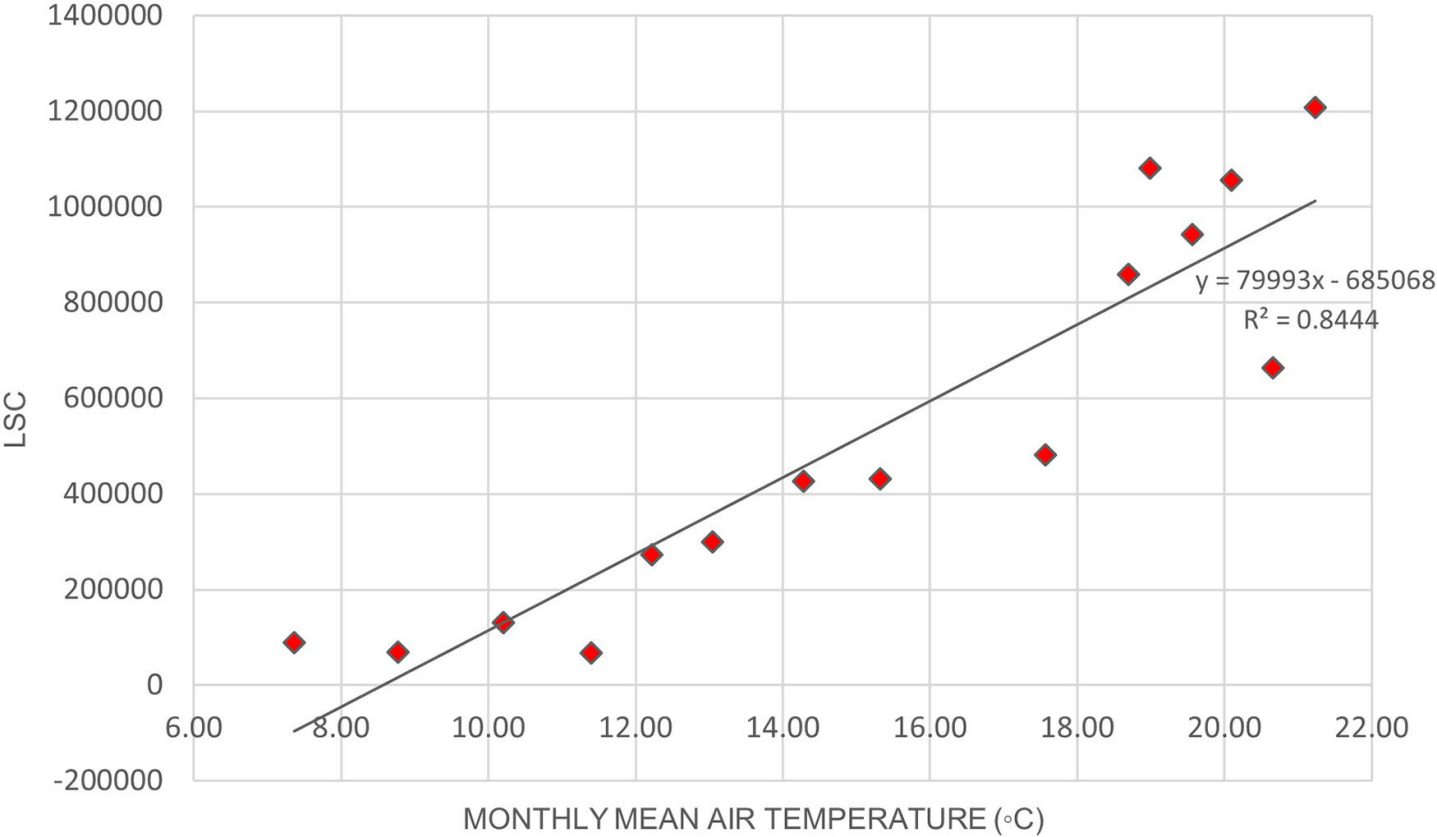
LSC vs air temperature over Europe during March to July 2020.

**Figure 3.**
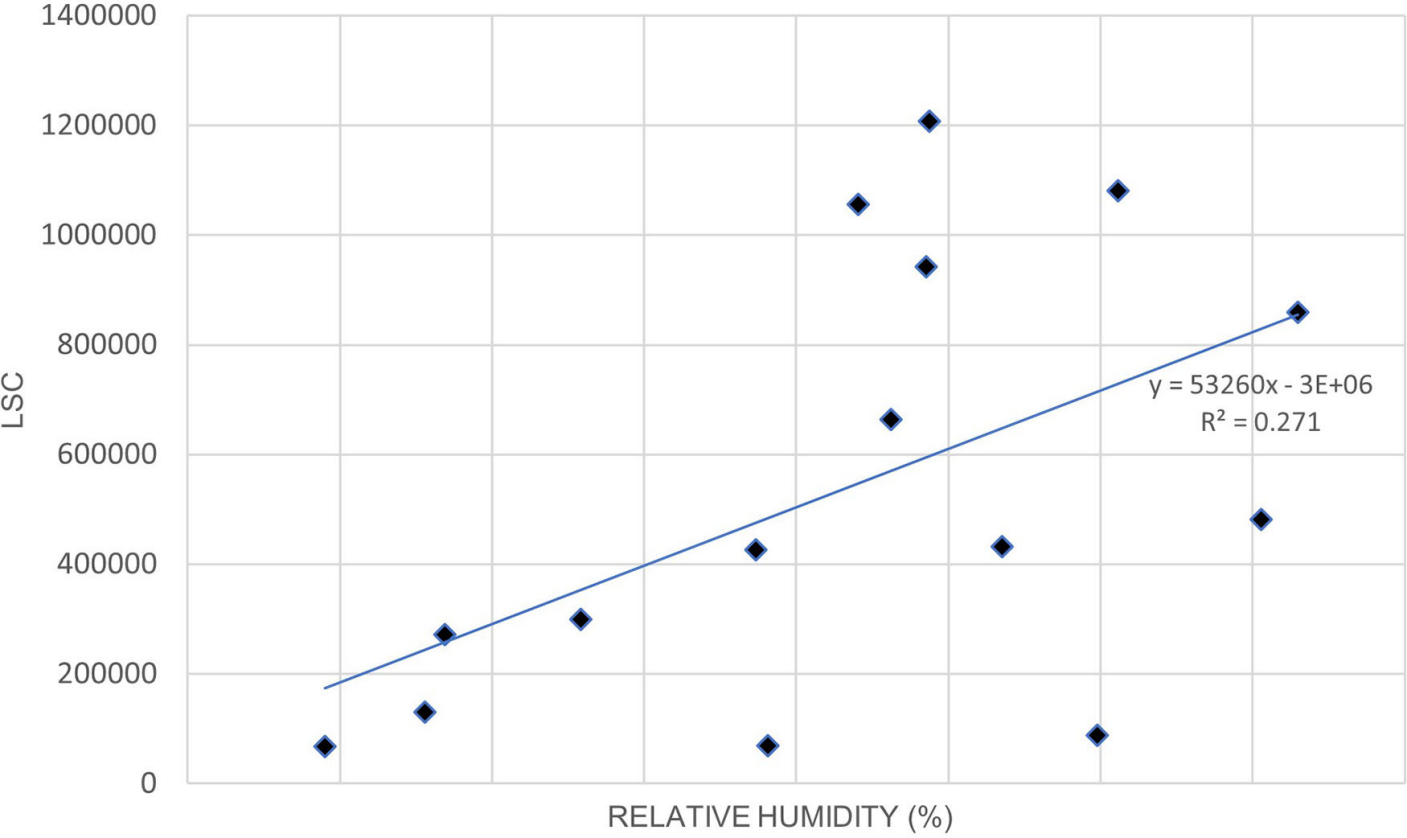
LSC vs relative humidity over Europe during March to July 2020.

Figure 2 illustrates the variation of LSC against air temperature levels in Europe. Figure 3. illustrates the relationship between LSC and relative humidity in Europe. Table 6 shows that the correlation of lightning strikes with air temperature and relative humidity in Europe are statistically significant. The Pearson correlation between lightning strikes and air temperature is 0.92, indicating a strong positive relationship between the variables. Pearson correlation between LSC and relative humidity is 0.52, indicating a moderate positive relationship between the variables. Higher relative humidity may enhance the upward updraft and easing the particle collision in the cloud. On the other hand, lower relative humidity may lead to weaker updraft and decreased the chance of lightning occurrence. The positive correlation between lightning strikes with air temperature and relative humidity in Europe concurs with the findings of. ^17, 19, 20, 28, 29^

**Table 6.** Correlation strength of lightning strikes with air temperature and relative humidity in Europe.

	Correlation coefficient, R	P-value	Correlation strength
Air temperature	0.92	<.001	Very high
Relative humidity	0.52	.047	Moderate

### Oceania

**Figure 4.**
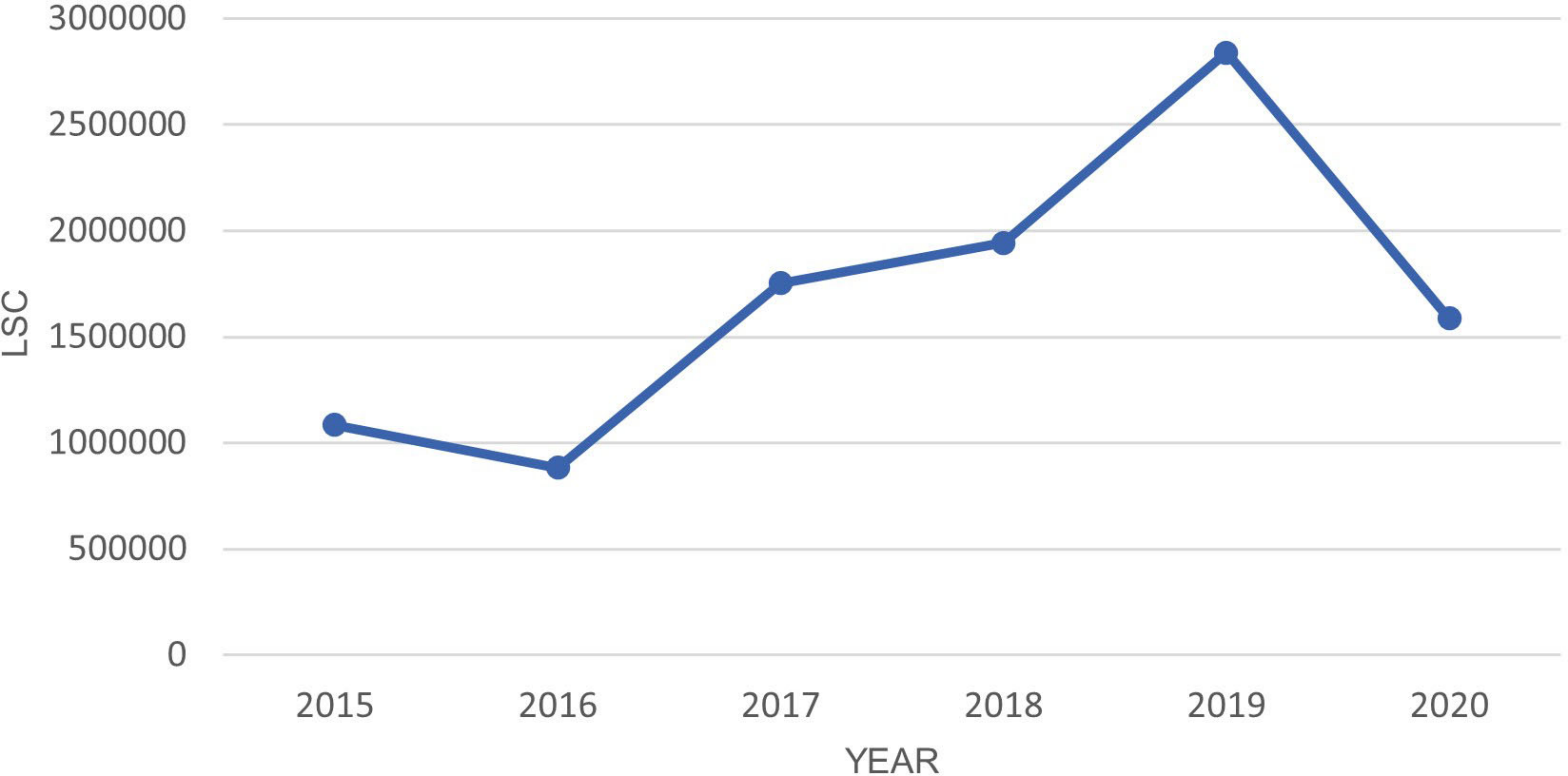
LSC in Oceania from March-July in year 2015–2020.

**Figure 5.**
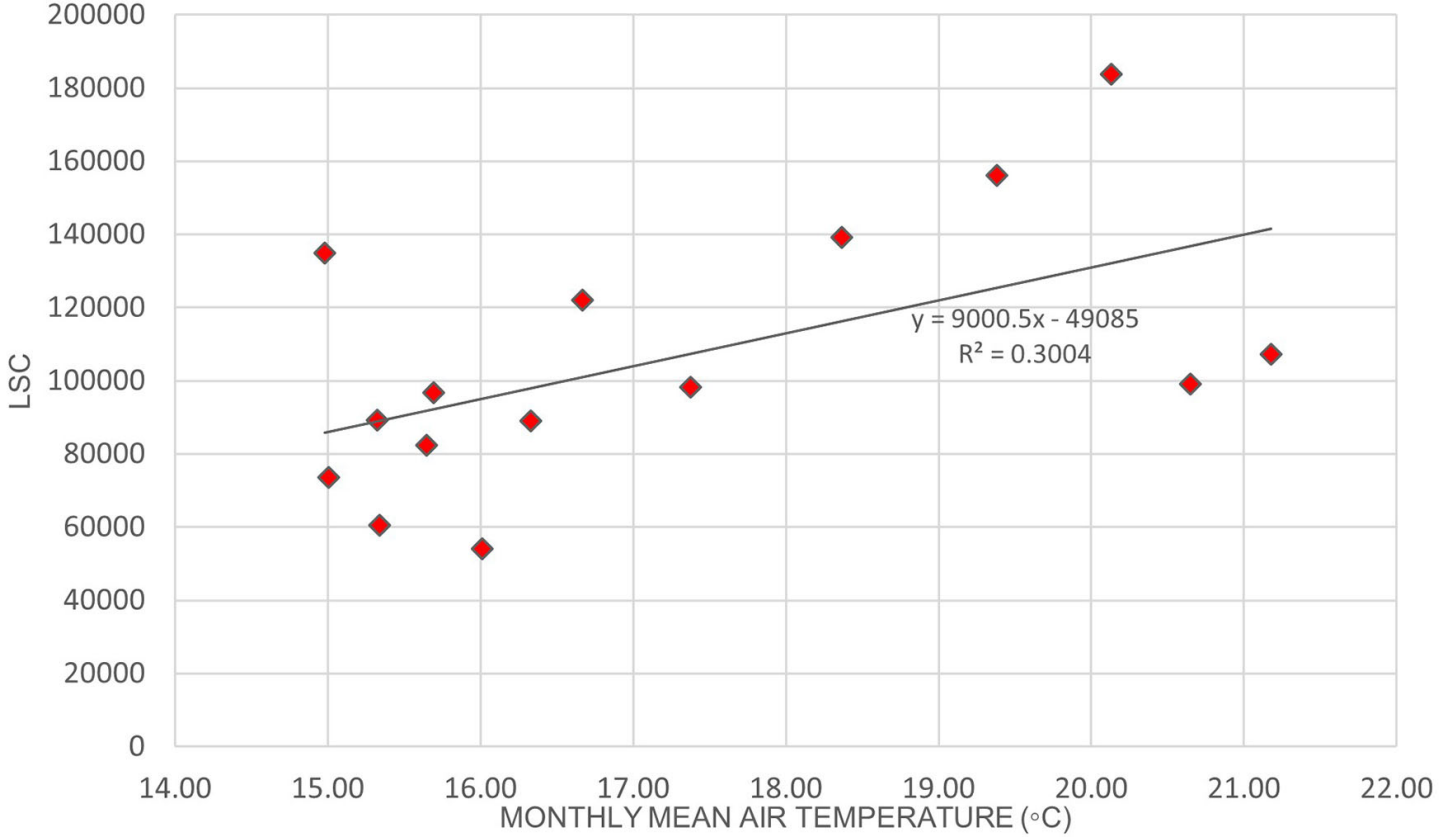
LSC vs air temperature over Oceania during March to July 2020.

**Figure 6.**
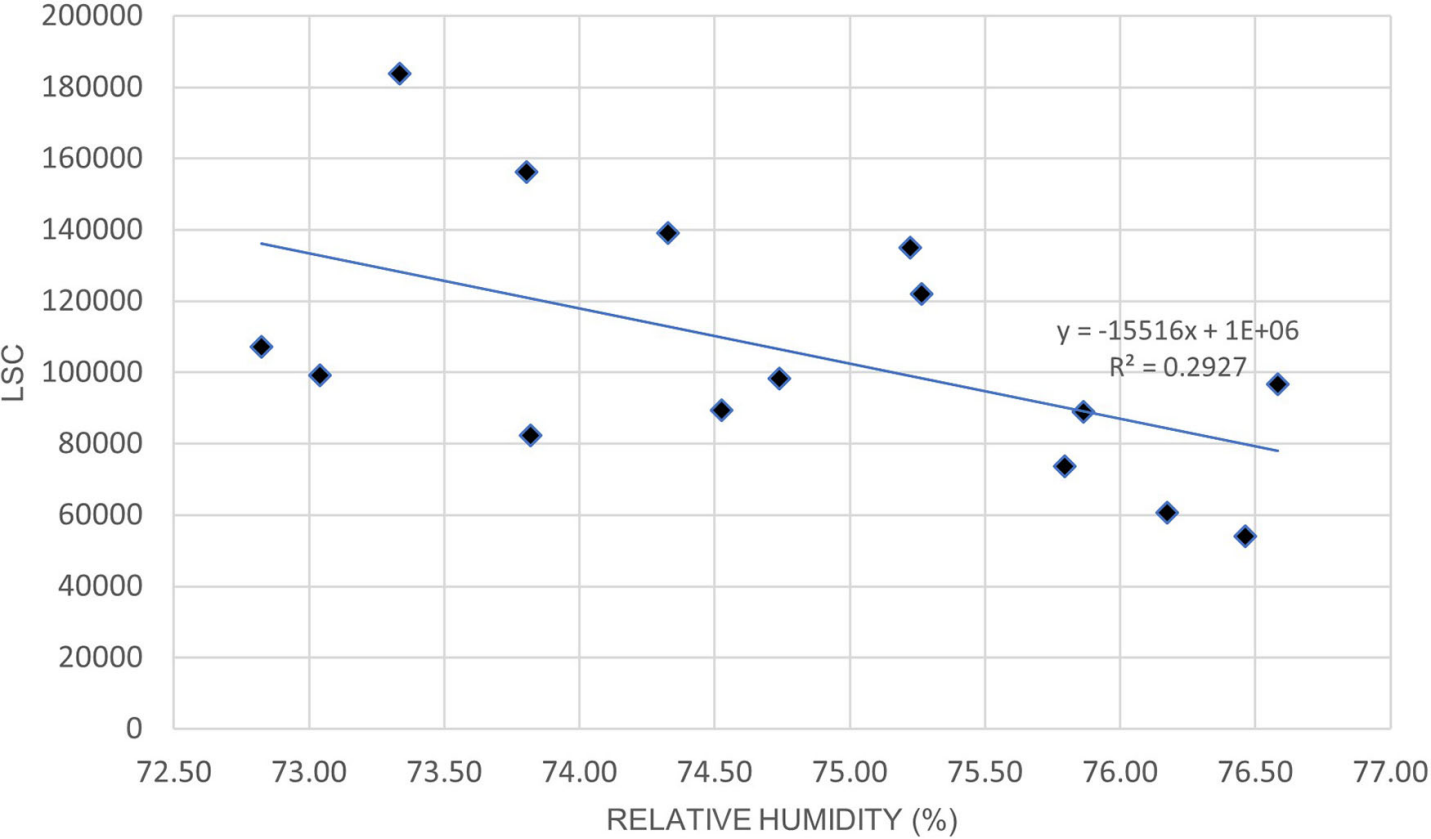
LSC vs relative humidity over Oceania during March to July 2020.

There was a 44% drop in LSC from 2019 to 2020 as shown in Figure 4. Table 7 shows there is statistically significant difference between the year 2020 with all previous years except 2017. Figure 5 and Table 8 indicates a moderate positive correlation between LSC and air temperature in Oceania during the lockdown period. Unlike Europe, Figure 6 and Table 8 shows that the relationship between LSC and relative humidity in Oceania is negatively correlated. The positive correlation of LSC and air temperature is consistent with previous studies. ^28, 29^ The negative correlation of LSC and relative humidity in Oceania obtained in this study contradicted the study of Shi et al. ^20^

**Table 7.** t-test results comparing lightning strikes in 2020 with previous years in Oceania.

**Comparison of lightning strikes in 2020 and 2019**			
**Year**	**Mean**	**P-value**	**Decision**
2020	105767	.016	Reject $H_{0}$
2019	189324		
**Comparison of lightning strikes in 2020 and 2018**			
**Year**	**Mean**	**P-value**	**Decision**
2020	105767	.050	Reject $H_{0}$
2018	129513		
**Comparison of lightning strikes in 2020 and 2017**			
**Year**	**Mean**	**P-value**	**Decision**
2020	105767	.536	Do Not Reject $H_{0}$
2017	116795		
**Comparison of lightning strikes in 2020 and 2016**			
**Year**	**Mean**	**P-value**	**Decision**
2020	105767	.001	Reject $H_{0}$
2016	58794		
**Comparison of lightning strikes in 2020 and 2015**			
**Year**	**Mean**	**P-value**	**Decision**
2020	105767	.012	Reject $H_{0}$
2015	72232		

**Table 8.** Correlation strength of lightning strikes with air temperature and relative humidity in Oceania.

	Correlation coefficient, R	P-value	Correlation strength
Air temperature	0.55	.034	Moderate
Relative humidity	−0.54	.037	Moderate

## Conclusions

In conclusion, there was a drastic drop in LSC in Europe and Oceania during the first lockdown period in 2020. A dependent t-test confirmed that a statistically significant difference in LSC between Period A and Period B. There is a positive relationship between LSC and air temperature in Europe (r = 0.92) and Oceania (r = 0.55). Furthermore, there is a positive relationship between LSC and relative humidity in Europe (r = 0.52) but a negative relationship between LSC and relative humidity in Oceania (r = −0.54).

The difference in correlation findings between lightning and relative humidity in Europe and Oceania remains unexplained. Higher relative humidity will lead to stronger updraft and increased lightning occurrence. However, too much vapor may weaken the updraft by blocking the vapor to rise up to complete the phase transformation.

The differences in correlation between lightning, air temperature, and relative humidity in Europe and Oceania may also be due to other possible factors such as aerosol level, wind motions, and particulate matter. Future work should be replicated in other geographical regions such as America and Asia.

## Author roles

Fazandra Y: Conceptualization, Formal Analysis, Methodology, Writing – Original Draft Preparation, Writing – Review & Editing;

Siow C.L.: Conceptualization, Supervision, Writing – Review & Editing

Chandima G.: Conceptualization, Writing – Review & Editing

Aravind C.: Methodology, Validation

Lee C.P.: Validation, Supervision

## Data availability statement

All data underlying the results are available as part of the article and no additional source data are required.
