# Supplementary material for: Lightning Behaviour during the COVID-19 Pandemic
Source: F1000Res. 2021 Nov 3;10:906. Originally published 2021 Sep 9. [Version 3] doi: 10.12688/f1000research.70650.3 (PMC8573680; doi:10.12688/f1000research.70650.3)
Supplement: Peer review history

# Peer review: Reviewer response for version 3

**DOI:** 10.5256/f1000research.78909.r98863

**Published:** 2021-11-04

**Authors:** Francisco Javier Pérez Invernón

**Affiliations:** 1 Deutsches Zentrum für Luft- und Raumfahrt, Institut für Physik der Atmosphäre, Oberpfaffenhofen, Germany

I thank the authors for taking into consideration my comments. I think the paper can be indexed in its present form.

Is the work clearly and accurately presented and does it cite the current literature?

Partly

If applicable, is the statistical analysis and its interpretation appropriate?

Partly

Are all the source data underlying the results available to ensure full reproducibility?

Yes

Is the study design appropriate and is the work technically sound?

Partly

Are the conclusions drawn adequately supported by the results?

No

Are sufficient details of methods and analysis provided to allow replication by others?

Yes

Reviewer Expertise:

Atmospheric electricity, meteorology, plasma physics.

I confirm that I have read this submission and believe that I have an appropriate level of expertise to confirm that it is of an acceptable scientific standard.

# Peer review: Reviewer response for version 2

**DOI:** 10.5256/f1000research.78244.r98046

**Published:** 2021-10-28

**Authors:** Francisco Javier Pérez Invernón

**Affiliations:** 1 Deutsches Zentrum für Luft- und Raumfahrt, Institut für Physik der Atmosphäre, Oberpfaffenhofen, Germany

The authors have successfully addressed most of my comments and the manuscript has improved.

 However, I still recommend adding some statement about the Detection Efficiency (DE) of the sensors used by LightningMaps.org. The authors assumed that the DE has remained constant between 2015 and 2020 and that the observed changes in the monthly total number of lightning flashes is not an artifact. If the authors cannot check this assumption, I think they should explicitly mention that. For example, they could simply mention that they assume that the DE of the sensors used by LightningMaps.org has remained constant during the investigated period.

Is the work clearly and accurately presented and does it cite the current literature?

Partly

If applicable, is the statistical analysis and its interpretation appropriate?

Partly

Are all the source data underlying the results available to ensure full reproducibility?

Yes

Is the study design appropriate and is the work technically sound?

Partly

Are the conclusions drawn adequately supported by the results?

No

Are sufficient details of methods and analysis provided to allow replication by others?

Yes

Reviewer Expertise:

Atmospheric electricity, meteorology, plasma physics.

I confirm that I have read this submission and believe that I have an appropriate level of expertise to confirm that it is of an acceptable scientific standard, however I have significant reservations, as outlined above.

# Response

**Published:** 2021-10-29

**Authors:** Chun Lim Siow

**Affiliations:** Multimedia University, Malaysia

Thank you for the recommendation to improve the quality of this manuscript.

 We have added the following sentence to clarify our assumption of Detection Efficiency:

*"It is assumed that the Detection Efficiency of the sensors used by LightningMaps.org remained constant during the period considered in this study."*

# Peer review: Reviewer response for version 1

**DOI:** 10.5256/f1000research.74252.r94274

**Published:** 2021-09-23

**Authors:** Wooi Chin Leong

**Affiliations:** 1 Centre of Excellence for Renewable Energy, School of Electrical Systems Engineering, Pauh Putra Campus, Universiti Malaysia Perlis, Arau, Malaysia

This is an interesting study on the lightning activities during the covid-19 pandemic periods. This article also relates lightning to air temperature and humidity. However, there are some parts of the article that can be further justified in order to improve the paper quality. Some suggestions have been made here:

1. What is the definition of the mean value shown in table 5?
2. Perhaps the authors can further explain the relationship between temperature and LSC. Why does low temperature mean less LSC etc?
3. Perhaps the authors can further explain the relationship between humidity and LSC. Why does low temperature mean less LSC etc?
4. A suggestion for the authors to discuss the relationship between months and lightning events/LSC, whether the lockdown or economic slow down affects the lightning occurrence. The authors may provide reasons to justify it as well.

Is the work clearly and accurately presented and does it cite the current literature?

Yes

If applicable, is the statistical analysis and its interpretation appropriate?

Yes

Are all the source data underlying the results available to ensure full reproducibility?

Yes

Is the study design appropriate and is the work technically sound?

Yes

Are the conclusions drawn adequately supported by the results?

Yes

Are sufficient details of methods and analysis provided to allow replication by others?

Yes

Reviewer Expertise:

Lightning

I confirm that I have read this submission and believe that I have an appropriate level of expertise to confirm that it is of an acceptable scientific standard.

# Response

**Published:** 2021-10-06

**Authors:** Chun Lim Siow

**Affiliations:** Multimedia University, Malaysia

**Comment:**

 This is an interesting study on the lightning activities during the covid-19 pandemic periods. This article also relates lightning to air temperature and humidity. However, there are some parts of the article that can be further justified in order to improve the paper quality. Some suggestions have been made here:

 What is the definition of the mean value shown in table 5?

**Response:**

 Thank you for the comment. It actually means the mean value of lightning strikes from March-July. We have added the following to clarify:

*Exact P values and the mean value of lightning strikes from May to July are provided in* *Table 5*

**Comment:**

 Perhaps the authors can further explain the relationship between temperature and LSC. Why does low temperature mean less LSC etc?

**Response:**

 Thank you for the comment. We have added the following explanation in paragraph 3:

*When warm, wet air rises into cold air, thunderstorms form. As the warm air cools, moisture in the form of water vapour condenses into water droplets, a process known as condensation. Cooled air descends through the atmosphere, warms up, and rises again. A convection cell is a circuit of rising and descending air. A cloud will form if this happens in a small amount. A thunderstorm can arise if this happens with a lot of air and moisture.*

*The presence of high air temperature can quickly rise and cause powerful updrafts. These updrafts carried water droplets and quickly freeze and begin collision with ice crystals and graupel which causing charge transfer process. Therefore, low temperature may slow down this process.*

**Comment:**

 Perhaps the authors can further explain the relationship between humidity and LSC. Why does low temperature mean less LSC etc?

**Response:**

 Thank you for the comment. Studies found that higher relative humidity may enhance the upward updraft and easing the particle collision in the cloud. Lower relative humidity may lead to weaker updraft and decreased the chance of lightning occurrence. This trend has been demonstrated in Europe but not in Oceania. We have added the explanation in the Results and Discussion section under Europe.

**Comment:**

 A suggestion for the authors to discuss the relationship between months and lightning events/LSC, whether the lockdown or economic slow down affects the lightning occurrence. The authors may provide reasons to justify it as well.

**Response:**

 Thank you for the suggestion. The introduction section has been further enhanced by incorporating additional references to support our argument.

# Peer review: Reviewer response for version 1

**DOI:** 10.5256/f1000research.74252.r94352

**Published:** 2021-09-16

**Authors:** Francisco Javier Pérez Invernón

**Affiliations:** 1 Deutsches Zentrum für Luft- und Raumfahrt, Institut für Physik der Atmosphäre, Oberpfaffenhofen, Germany

This manuscript reports an analysis of the changes in lightning, temperature and relative humidity between 2015 and 2020, including the COVID-19 lockdown period. The authors find a significant change in these variables during the lockdown. They perform a statistical analysis to find possible relationships between the temperature, relative humidity and lightning. They find a possible correlation between them.

 The analysis of these variables is interesting, as well as finding possible relationships between them. However, the authors do not report a solid connection between the COVID-19 pandemic and the variables.

 The topic is in the scope of F1000 Research. The manuscript is well written and organized. However, references are not updated, the method needs some more clarification, and the conclusions are not supported by the results. In addition, I miss a Discussion.

 I think the authors have to make a major revision before this manuscript can be accepted for indexing. If the authors cannot explain the possible relationship between the reduction of lightning and the pandemic, they may consider excluding the attribution of the decrease in lightning activity to the pandemic.

 I provide specific comments below:

**Introduction**

 Second paragraph: Please mention the reduction in aerosols during the lockdown.

 The reference provided by the authors for the relationship between CO2 emissions and temperature reports a significant positive correlation between the atmospheric temperature and CO2 emission at a scale of years. Please provide some reference for a possible correlation between CO2 and temperature at a time period similar to the lockdown (weeks-months). Jones *et al.* (2021) ^1^ did not find any relationship between the CO2 reduction and changes in temperature.

 Third paragraph: Some authors have reported a possible decrease in lightning activity due to climate change (Finney *et al., *2018) ^2^.

 Fourth paragraph: Please explain how aerosols are involved in lightning activity.

 "Lightning ground flash density tends to increase with drier and warmer surface air": Please provide some reference. In general, lightning does not tend to increase with drier surface.

 Why mention Denver and Colorado in this study?

 Previous studies have already investigated the possible relationship between the lockdown and lightning activity. Please cite and explain them in the introduction: Chowdhuri *et al.* (2020) ^3^, Pinto Neto *et al.* (2020) ^4^, Pérez-Invernón *et al.* (2021) ^5^.

**Methods**

 The authors have to estimate the temporal evolution of the lightning Detection Efficiency of the sensors used by LightningMaps.org, as you will study the temporal evolution of lightning in the data set. Lightning can increase or decrease year by year due to changes in the Detection Efficiency. The Detection Efficiency of the data set can be investigated by comparison with other lightning data set, such as LIS.

**Results**

 Figures 2, 3, 5 and 6: What does each point represent?

**General comments and conclusions**

 This study shows that there could be a possible relationship between temperature, relative humidity and lightning in the studied regions (Europe and Oceania). However, the influence of the COVID-19 pandemic is not clear. There were significant changes in both temperature and relative humidity during the pandemic that could influence lightning. However, such changes could not be connected to the pandemic. See for example Jones *et al.* (2021) ^1^, who used 12 models to produced over 300 simulations. They did not find any associated impact of the reduction of CO2 on temperature or rainfall. Changes observed in temperature and relative humidity can be due to other factors instead of the pandemic.

 In addition, the authors find an opposite correlation between relative humidity and lightning in Europe and Oceania. The reasons for this opposite relationship are not explained.

 Finally, previous studies have found a connection between the reduction of lightning and the reduction in the concentration of aerosols during the pandemic. However, this study does not investigate the role of aerosols in lightning activity.

Is the work clearly and accurately presented and does it cite the current literature?

Partly

If applicable, is the statistical analysis and its interpretation appropriate?

Partly

Are all the source data underlying the results available to ensure full reproducibility?

Yes

Is the study design appropriate and is the work technically sound?

Partly

Are the conclusions drawn adequately supported by the results?

No

Are sufficient details of methods and analysis provided to allow replication by others?

Yes

Reviewer Expertise:

Atmospheric electricity, meteorology, plasma physics.

I confirm that I have read this submission and believe that I have an appropriate level of expertise to state that I do not consider it to be of an acceptable scientific standard, for reasons outlined above.

## References

[1] : The Climate Response to Emissions Reductions Due to COVID-19: Initial Results From CovidMIP. *Geophys Res Lett* .2021;48(8) : 10.1029/2020GL091883 e2020GL091883 10.1029/2020GL091883 34149115PMC8206678

[2] : A projected decrease in lightning under climate change. *Nature Climate Change* .2018;8(3) : 10.1038/s41558-018-0072-6 210-213 10.1038/s41558-018-0072-6

[3] : Significant decrease of lightning activities during COVID-19 lockdown period over Kolkata megacity in India. *Sci Total Environ* .2020;747: 10.1016/j.scitotenv.2020.141321 141321 10.1016/j.scitotenv.2020.141321 32771791PMC7385625

[4] : Lightning during the COVID-19 pandemic in Brazil. *J Atmos Sol Terr Phys* .2020;211: 10.1016/j.jastp.2020.105463 105463 10.1016/j.jastp.2020.105463 33046961PMC7539900

[5] : Influence of the COVID-19 lockdown on lightning activity in the Po Valley. *Atmospheric Research* .2021;263: 10.1016/j.atmosres.2021.105808 10.1016/j.atmosres.2021.105808

# Response

**Published:** 2021-10-06

**Authors:** Chun Lim Siow

**Affiliations:** Multimedia University, Malaysia

**General Comments:**

 This manuscript reports an analysis of the changes in lightning, temperature and relative humidity between 2015 and 2020, including the COVID-19 lockdown period. The authors find a significant change in these variables during the lockdown. They perform a statistical analysis to find possible relationships between the temperature, relative humidity and lightning. They find a possible correlation between them.

 The analysis of these variables is interesting, as well as finding possible relationships between them. However, the authors do not report a solid connection between the COVID-19 pandemic and the variables.

 The topic is in the scope of F1000 Research. The manuscript is well written and organized. However, references are not updated, the method needs some more clarification, and the conclusions are not supported by the results. In addition, I miss a Discussion.

 I think the authors have to make a major revision before this manuscript can be accepted for indexing. If the authors cannot explain the possible relationship between the reduction of lightning and the pandemic, they may consider excluding the attribution of the decrease in lightning activity to the pandemic.

**Response to General Comments:**

 Thank you for the comment. The focus of this manuscript is more on examining any statistical relationship between the lightning activities and the changes in atmospheric temperature and humidity during the pandemic. Discussion is partially included with the results and in the conclusion. At this point of research, we could only discuss it statistically and refer to any concurring literature. We have addressed the comments and incorporated the recommendations at our level best.

 I provide specific comments below:

**Comment: Introduction**

 The reference provided by the authors for the relationship between CO2 emissions and temperature reports a significant positive correlation between the atmospheric temperature and CO2 emission at a scale of years. Please provide some reference for a possible correlation between CO2 and temperature at a time period similar to the lockdown (weeks-months). Jones *et al.* (2021) did not find any relationship between the CO2 reduction and changes in temperature.

**Response:**

 Thank you for the comment. The recommended reference has been cited. However, we could not find any reference for similar time period to the lockdown. Here is our response:

*The release of carbon dioxide (CO2) from the combustion of coal, oil, and natural gas is the primary driver of rising global temperatures. These activities, as can be seen, tend to decrease during the lockdown period. As a result, the rate of temperature rise may be delayed during the lockdown period. This, however, is insufficient to cause long-term effects on CO2 levels and climate change on a worldwide scale.*

**Comment:**

 Third paragraph: Some authors have reported a possible decrease in lightning activity due to climate change (Finney *et al., *2018).

**Response:**

 Thank you for the comment. The recommended reference has been cited. Here is our response:

*(Finney et al., 2018) stated that many previous studies found a positive correlation between lightning and temperature, and one previous study that found lightning decreases with an increase in temperature. This may explain that such relationships become highly uncertain on longer timescales.*

**Comment:**

 Fourth paragraph: Please explain how aerosols are involved in lightning activity.

**Response:**

 Thank you for the comment. The following has been added into fourth paragraph:

*Aerosol could affect lightning activity through modification of cloud micro-physics.*

*Aerosol particles serve as cloud condensation nuclei and ice nuclei, and the amount of this particles could affect the formation of cloud droplets and ice particles. More aerosol will suppress the coalescence and making the average size of cloud droplet to be reduced as well as inhibiting precipitation. Therefore, the process enables the water droplets to rise further to upper layers of the clouds and may enhance the lightning processes.*

**Comment:**

 "Lightning ground flash density tends to increase with drier and warmer surface air": Please provide some reference. In general, lightning does not tend to increase with drier surface.

**Response:**

 Thank you for the comment. We have provided reference based on the study from Diaz-Ortiz reference number 17 into third paragraph:

*Yes, in general lightning does not tend to increase with warmer and drier surface, but the presence of high air temperature can quickly rise and cause powerful updrafts. These updrafts carried water droplets and quickly freeze and begin collision with ice crystals and graupel which causing charge transfer process. Therefore, low temperature may slow down this process. So high temperature can be a driving factor to lightning formation.*

**Comment:**

 Why mention Denver and Colorado in this study?

**Response:**

 Thank you for the comment.

*Denver and Colorado have stated that lightning peaked during summer time, which may possibly explain the high temperature leads to high lightning activity.*

**Comment:**

 Previous studies have already investigated the possible relationship between the lockdown and lightning activity. Please cite and explain them in the introduction: Chowdhuri *et al.* (2020), Pinto Neto *et al.* (2020), Pérez-Invernón *et al.* (2021).

**Response:**

 Thank you for the comment. All the recommended articles are relevant and have been cited and explained accordingly.

**Comment: Methods**

 The authors have to estimate the temporal evolution of the lightning Detection Efficiency of the sensors used by LightningMaps.org, as you will study the temporal evolution of lightning in the data set. Lightning can increase or decrease year by year due to changes in the Detection Efficiency. The Detection Efficiency of the data set can be investigated by comparison with other lightning data set, such as LIS.

**Response:**

 Thank you for the comment. Currently, this is beyond our scope of work. We will consider this for our future work.

**Comment: Results**

 Figures 2, 3, 5 and 6: What does each point represent?

**Response:**

 Thank you for the comment. Each point represents the lightning strikes count (LSC). Figure 2 and 5 is the scatter plots of the LSC against air temperature. Figure 3 and 6 is the scatter plots of the LSC against humidity.

**General comments and conclusions**

 This study shows that there could be a possible relationship between temperature, relative humidity and lightning in the studied regions (Europe and Oceania). However, the influence of the COVID-19 pandemic is not clear. There were significant changes in both temperature and relative humidity during the pandemic that could influence lightning. However, such changes could not be connected to the pandemic. See for example Jones *et al.* (2021), who used 12 models to produced over 300 simulations. They did not find any associated impact of the reduction of CO2 on temperature or rainfall. Changes observed in temperature and relative humidity can be due to other factors instead of the pandemic.

**Response:**

 Thank you for the comment. We acknowledged the findings by Jones *et al*. (2021) as highlighted. However, we have supplemented our argument with a few supporting references.

**Comment:**

 In addition, the authors find an opposite correlation between relative humidity and lightning in Europe and Oceania. The reasons for this opposite relationship are not explained.

**Response:**

 Thank you for the comment. Unfortunately, it is beyond our current level of understanding to provide the exact explanation on the opposite relationship found.

*Higher relative humidity will lead to stronger updraft and increased lightning occurrence. However, too much vapor may weaken the updraft by blocking the vapor to rise up to complete the phase transformation.*

**Comment:**

 Finally, previous studies have found a connection between the reduction of lightning and the reduction in the concentration of aerosols during the pandemic. However, this study does not investigate the role of aerosols in lightning activity.

**Response:**

 Thank you for the comment. Yes, we have not considered in this in this study. However, we have noted your recommendation and have since enhanced the literature review of this study based on your feedback. We will consider the role of aerosols in lightning activity in our future work.
